# Faculty Changes

**Published:** 1882-05

**Authors:** J. B. Hodgkin


J. B. Hodgkin.

---

*Faculty Changes.*—As seen elsewhere two vacancies in the Faculty of the Baltimore College of Dental Surgery have been created by resignation. Prof. F. J. S. Gorgas, for many years the Dean of that college, has accepted the position of Dean of the newly created Dental Department of the University of Maryland. Dr. James H. Harris, Professor of Clincal Dentistry goes also to that School with Prof. Gorgas.

The vacancy in the old college created by the resignation of Prof. Gorgas, so far as relates to the office of Dean, has been filled by the election of Dr. Richard B. Winder, to that position. Dr. M. Whilldin Foster, is elected Prof. of Dental Pathology and Therapeutics, and Dr. J. E. Lindsay, Prof. of Chemistry. Dr. J. Emory Scott, is elected Demonstrator of Mechanical Dentistry, vice Dr. J. C. Uhler resigned.

All letters intended for the Baltimore College of Dental Surgery, will be addressed to Dr. Richard B. Winder, 140 Park Avenue, Baltimore, Md.

